# Effects of inoculation of *Lactiplantibacillus plantarum* and *Lentilactobacillus buchneri* on fermentation quality, aerobic stability, and microbial community dynamics of wilted *Leymus chinensis* silage

**DOI:** 10.3389/fmicb.2022.928731

**Published:** 2022-07-29

**Authors:** Baiyila Wu, Zongfu Hu, Manlin Wei, Mei Yong, Huaxin Niu

**Affiliations:** College of Animal Science and Technology, Inner Mongolia Minzu University, Tongliao, China

**Keywords:** *Leymus chinensis* silage, fermentation quality, microbial community, aerobic stability, *Lactiplantibacillus plantarum*, *Lentilactobacillus buchneri*

## Abstract

*Leymus chinensis* is an important crop that can be fed to ruminants. The purpose of this study was to investigate the roles of *Lactiplantibacillus plantarum* and *Lentilactobacillus buchneri* in fermentation quality, aerobic stability, and dynamics of wilted *L. chinensis* silage microorganisms. Wilted *L. chinensis* silages were ensiled with/without *L. plantarum* and *L. buchneri*. After 14 and 56 days of ensiling, the silos were opened and subjected to a 7-day aerobic deterioration test. This study looked at the composition of fermentation products as well as the microbial communities in silage. Silage inoculated with *L. plantarum* and *L. buchneri* had an increased lactic acid content as well as lactic acid bacterial (LAB) quantity, but a decrease in pH and levels of butyric acid, 2,3-butanediol, and ethanol was observed during ensiling. Non-treated and *L. plantarum*-treated silages deteriorated in the 7-day spoilage test after opening day-14 silos, whereas *L. buchneri*-inoculated silage showed no signs of deterioration. *Lactobacillus* abundance increased in the 7-day spoilage test after opening day-56 silos, while undesirable microorganisms such as *Acetobacter*, *Bacillus*, and molds, namely, *Aspergillus* and *Penicillium* were inhibited within *L. plantarum*- and *L. buchneri*-inoculated silages. The composition of fermentation products was related to the bacterial community, particularly *Lactobacillus*, *Enterococcus*, and *Acetobacter*. To summarize, *L. plantarum*- and *L. buchneri*-inoculated silage enhanced fermentation quality during ensiling and inhibited aerobic spoilage in a 7-day spoilage test of 56 days ensiling within wilted *L. chinensis* silage.

## Introduction

*Leymus chinensis* (Trin.) Tzvel. is a Gramineae perennial grass with a wide range of adaptations that is a major source of animal feed in China (Li et al., 2019). Due to its excellent palatability, tender stems and leaves, and capacity to meet ruminant nutrition demands, *L. chinensis* is one of the most important forage grasses in China’s Inner Mongolian Plateau, Korea, Russia, the Northern Plain, and Mongolia (Peng et al., 2013; Zhu et al., 2020). Despite its high yield in these areas, *L. chinensis* has a low lactic acid bacteria (LAB) count and water-soluble carbohydrates (WSCs) level, which can hinder ensilage as a method of grass conservation (Zhang et al., 2014). Forages must develop an appropriate ensilage process because feeding high-quality silage is supposed to increase the productivity of livestock such as beef and dairy cattle.

Lactic acid bacteria inoculants have been widely used to stimulate the fermentation process in order to produce high-quality silage. Previous studies have reported the classification of inoculants as homofermentative and heterofermentative bacteria based on fermentation profiles (Muck et al., 2018). Many homofermentative LAB strains, such as *Lactiplantibacillus plantarum*, *Pediococcus* species, and *Enterococcus faecium*, have been widely used for dominating ensiling fermentation by rapidly producing lactic acid while lowering pH, preventing yeast, mold, and other undesirable microorganisms, and preserving forage nutrients during storage (Ranjit and Kung, 2000). However, aerobic spoilage of sorghum, wheat, corn, and Italian ryegrass silages is increased when homofermentative LAB is used because the elevated lactic acid is rapidly oxidized by yeasts and other aerobic microorganisms after silage air exposure (Weinberg et al., 2002; Tabacco et al., 2009; Li and Nishino, 2011a). One of the heterofermentative LAB species, *Lentilactobacillus buchneri*, was developed as a silage inoculant to prevent aerobic degradation. Under anaerobic conditions, *L. buchneri* inhibited aerobic deterioration by fermenting lactic acid into 1,2-propanediol and acetic acid (Oude Elferink et al., 2001). A large amount of acetic acid was produced to protect the silage from aerobic molds and yeasts that cause aerobic spoilage (Nishino et al., 2003).

Over the last few decades, culture-dependent/independent approaches, such as 16S-rRNA gene sequences, real-time PCR, ribosomal intergenic spacer analysis, and characterizing denaturing gradient gel electrophoresis bands, have been used to investigate the epiphytic microbiota in pre-ensiled crops and the microbiota in ensiled forages (Ennahar et al., 2003; Stevenson et al., 2006; Brusetti et al., 2011; Wu et al., 2014). Although the preceding studies provide information on microorganism composition in silage processes, the associated analyses only discovered a few major operational taxonomic units (OTUs) and failed to provide detailed information on microbial community composition (Ni et al., 2017). Next-generation sequencing (NGS; e.g., the ITS unit for fungi and 16S rRNA for bacteria) has proven to be a quick and accurate molecular tool for defining silage ecology, such as a description of microorganisms within silage (Wang et al., 2020b).

Furthermore, Zhang et al. (2016) discovered dynamic changes in bacterial communities in *L. chinensis* at different stages of silage and after aerobic exposure. To the best of our knowledge, however, only a few studies have looked at dynamic changes in fungal populations during *L. chinensis* silage and after aerobic exposure. This study looked at how microbial quantities, fermentation products, and Illumina MiSeq sequencing changed during the silo opening and the aerobic deterioration test in wilted *L. chinensis* inoculated with *L. plantarum* and *L. buchneri*. This study sought to determine whether changes in microbial communities could account for changes in aerobic stability and ensiling fermentation.

## Materials and methods

### Ensiling and bacterial inoculants

At the boot stage on 29 July 2021, *L. chinensis* was collected from an experimental field at China’s Inner Mongolia Minzu University. After a 5-h wilting period, the grass was chopped to an estimated particle length of 20 mm with a crop cutter to achieve the desired dry matter (DM) of 436 g/kg. Both *L. plantarum* (No. 6026) and *L. buchneri* (No. 20294) were successfully inoculated in *L. chinensis* silage at 1 × 10^6^ colony-forming unit (CFU)/g of fresh matter (FM). For the control group, the same amount of distilled water was sprayed. Each 300 g of non-inoculated and inoculated materials were packed tightly into plastic pouches (BN-10, 250 × 350 mm; Wangnuo, Beijing, China) using a commercial vacuum sealer (ZK-320; Ouxin, Beijing, China). Triplicate silages were prepared for each treatment, and silos were stored at room temperature for 14 and 56 days, respectively.

### Aerobic spoilage

After opening, 150 g of silage (half the amount) was placed in a 500-ml polyethylene (PE) bottle without being compacted. These uncapped bottles were exposed to air for 7 days at 25°C.

### Chemical analysis and microbial enumeration

For pH and organic acid determination, each fresh sample (20 g) was homogenized with sterilized water (180 ml) within the blender for 1 min, followed by filtration using a membrane (0.22 μm). The pH of this extract was determined immediately using the glass electrode pH meter (SX-620, Sanxin, Shanghai, China). An ion-exclusion polymeric high-performance liquid chromatography (HPLC) equipped with an refractive index (RI) detector was used to evaluate the composition of fermented products (Wu and Nishino, 2016).

The fresh material was oven-dried under 65°C for 48 h to obtain DM content. After that, each sample was grounded in a Wiley mill with a 1-mm sieve (ZM200, Retsch GmbH). The standard Association of Official Analytical Chemists (AOAC, 1990) procedures were utilized for measuring crude protein (CP) amount. Acid detergent fiber (ADF) and neutral detergent fiber (NDF) were assessed using Van Soest et al.’s (1991) method, whereas WSCs were quantified using the phenol-sulfuric acid assay (Wu and Nishino, 2016).

A serial dilution (from 10^–1^ to 10^–6^) was performed on a clean bench. To count LAB, de Man, Rogosa, and Sharpe agar (CM0361B, Oxoid Ltd., United Kingdom) was used, while violet red bile agar (CM0107B, Oxoid Ltd., United Kingdom) was used for enterobacterial counting. Molds and yeasts were quantified using spread plates in potato dextrose agar (CM0139B, Oxoid Ltd., United Kingdom) at pH 3.5 (maintained with sterile lactic acid). Colony counts in CFU/g of FM were used to calculate the viable microbial number.

### Microbial community analysis

The refrigerated silage (10 g) was blended for 2 h at 120 rpm with sterile PBS (40 ml; pH 7.4) using an electronic oscillator. The sample was then filtered using double-gauze masks. After 10 min of centrifugation of the filtrate at 4°C and 13,000 × *g*, the supernatant was removed and the pellet was kept on dry ice. Majorbio Bio-Pharm Technology in Shanghai, China, was used for metagenomic sequencing, which included PCR amplification and DNA extraction, followed by Illumina MiSeq sequencing and final sequencing data processing. The UPARSE version 7.1 (Edgar, 2013) was used to cluster the OTUs at the 97% similarity threshold. Following the identification and elimination of Chimeric sequences, the RDP Classifier (version 2.2) was used for the taxonomic analysis of typical OTU sequences against a 16S rRNA database such as Silva version 138, with a confidence level of 0.7 (Yang et al., 2019). The free and open Majorbio Cloud Platform was used to analyze sequencing data from fungi and bacteria.

### Statistical analyses

The data were analyzed statistically using JMP version 13 software (SAS Institute, Japan) and bidirectional ANOVA, with inoculation and ensilage duration as primary factors. To assess the inoculation effect, the unidirectional ANOVA and subsequent Tukey’s test-based multiple comparisons were used. To determine significance, *P* < 0.05 was used.

## Results and discussion

### Microbial and chemical compositions of pre-ensiled *Leymus chinensis*

Table 1 shows microbial and chemical compositions of pre-ensiled *L. chinensis*. DM level in *L. chinensis* was 436 g/kg of FM, which was comparatively higher than that of tropical legume and grass silages (Heinritz et al., 2012). CP level was found to be 93.71 g/kg of DM, which is greater than the 84.40 g/kg of DM found by Zhang et al. (2016). WSC served as the critical substrate in silage fermentation, with a level of greater than 60 g/kg of DM ensuring the desired grade of fermentation. This study determined WSC as 32.58 g/kg of DM in pre-ensiled *L. chinensis*, indicating that it was difficult to achieve good preservation without adding anything during the ensilage process. The epiphytic LAB count was 3.67 log CFU/g of FM, yeast count was 5.47 log CFU/g of FM, and enterobacterial count was 5.12 log CFU/g of FM. The epiphytic LAB count on fresh crops was usually considered an important factor in deciding whether bacterial inoculants should be applied in silage materials and predicting silage fermentation quality. The LAB count on forage material must be above 5.00 log CFU/g of FM to ensure a successful ensilage (Cai et al., 1998). The *L. chinensis* silage fermentation quality was improved by introducing LAB inoculants because of the low LAB count and high harmful bacteria count.

**TABLE 1:** Chemical and microbial compositions of pre-ensiling *Leymus chinesis*.

	Wilted *Leymus chinesis*
Dry matter (g/kg)	436 ± 6.12
pH	5.74 ± 0.31
Crude protein (g/kg DM)	93.71 ± 1.78
Neutral detergent fiber (g/kg DM)	612.32 ± 23.63
Acid detergent fiber (g/kg DM)	287.36 ± 5.76
Hemicellulose (g/kg DM)	325.32 ± 7.33
Acid detergent lignin (g/kg DM)	27.61 ± 0.66
Water-soluble carbohydrate (g/kg DM)	32.58 ± 0.92
Lactic acid bacteria (log cfu/g)	3.67 ± 0.32
Yeasts (log cfu/g)	5.47 ± 0.18
Enterobacteria (log cfu/g)	5.12 ± 0.36

Data are mean of duplicate analyses.

### Roles of *Lactiplantibacillus plantarum* and *Lentilactobacillus buchneri* in silage quality during ensiling and after aerobic exposure

According to Table 2, the lactic acid level was 8.33 g/kg of DM on the 14th day for the untreated silage, while the acetic acid level was 3.38 g/kg of DM. Both contents increased when ensilage in untreated silage was prolonged, but propionic acid was not detected at 14 or 56 days. The content of ethanol was 3.42 g/kg of DM, while the content of 2,3-butanediol was 2.66 g/kg of DM. It was reported that 2,3-butanediol can be produced from sugar by yeasts, *Bacillus* spp., and enterobacteria, and citric and malic acids by the two types of LAB (Mcdonald et al., 1991). *L*. *plantarum*, a homofermentative LAB species, was previously used as an additive to improve alfalfa fermentation quality (Filya et al., 2007; Contreras-Govea et al., 2013; Zhang et al., 2015). In comparison with the untreated control, inoculation with *L. plantarum* increased LAB and lactic acid and decreased pH and levels of butyric acid, 2,3-butanediol, and ethanol. Heterofermentative LAB-treated silages had higher levels of lactic acid, 1,2-propanediol, and acetic acid (Li and Nishino, 2011a). In this study, *L. buchneri* inoculation increased lactic acid, acetic acid, 1,2-propanediol, and LAB number while decreasing pH, butyric acid, 2,3-butanediol, and ethanol content. Regardless of the ensiling process, enterobacteria could become dominant in untreated silage. However, because of the rapid acidification and antagonistic effects of *L. plantarum* and *L. buchneri*, the enterobacterial count rapidly decreased, and the ethanol production efficiency was limited.

**TABLE 2:** Fermentation products and microbial counts of wilted *Leymus chinesis* silage without and with *Lactiplantibacillus plantarum* (LP) or *Lentilactobacillus buchneri* (LB) and subjected to 14 and 56 days of preservation.

	14 days				56 days				2-way ANOVA		
					
	C	LP	LB	SE	C	LP	LB	SE	I	S	I × S
Dry matter (g/kg)	425	446	432	5.23	448	425	433	7.99	NS	NS	*
pH	5.35a	4.54c	4.73b	0.05	4.62x	4.15y	4.30y	0.05	**	**	*
Lactic acid (g/kg DM)	8.33c	14.6a	12.2b	0.43	11.5z	18.2x	13.4y	0.47	**	**	**
Acetic acid (g/kg DM)	3.38b	4.61b	6.67a	0.33	4.25y	3.62z	6.33x	0.06	**	NS	**
Butyric acid (g/kg DM)	2.57a	0.26c	0.78b	0.06	1.20x	0.49y	0.36y	0.09	**	**	NS
Ethanol (g/kg DM)	3.42a	1.73c	2.64b	0.05	1.73x	0.24y	0.27y	0.05	**	**	**
2,3-Butanediod (g/kg DM)	3.62a	0.83b	1.62b	0.07	4.70x	1.68z	2.65y	0.11	**	**	**
1,2-Propanediol (g/kg DM)	0.56b	0.82b	2.23a	0.09	1.27y	1.51y	2.14x	0.09	**	**	**
Lactic acid bacteria (log cfu/g)	7.47b	9.44a	9.58a	0.10	7.31y	8.49x	8.51x	0.10	**	**	**
Yeasts (log cfu/g)	8.46	<2.00	<2.00	–	9.50	<2.00	<2.00	–	–	–	–
Enterobacteria (log cfu/g)	7.45	<2.00	<2.00	–	6.62	<2.00	<2.00	–	–	–	–

Means from triplicate silages; and Values for identical preservation periods with varying following letters (a-c, x-z) differ significantly (P < 0.05). I and S, respectively, represent the effects of inoculations and preservation periods, and I × S refers to the interaction of the two. **P < 0.01; *P < 0.05; and NS indicates P ≥ 0.05.

### Roles of *Lactiplantibacillus plantarum* and *Lentilactobacillus buchneri* in aerobic stability

Heating was observed on the third day after silo opening for *L. plantarum*-treated and non-inoculated silages, which were opened on the 14th day. The pH and yeast counts of the *L. plantarum*-treated silages were raised to > 6.0 and > 10^8^ CFU/g, respectively (Table 3). However, no heating was observed, followed by aerobic exposure in *L. buchneri*-treated silages opened on the 14th day, and significantly higher acetic acid and 1,2-propanediol contents were observed in the 7-day spoilage test after silo opening. In the 56-day non-inoculated silage, heating was observed on the 5th day after silo opening because yeast quantities exceeded 10^8^ levels in the 7-day spoilage test. Yeast counts exceeded 10^5^, indicating that the aerobic stability of the silage had been compromised (Mcdonald et al., 1991). Whole crop corn silage showed decreasing aerobic stability after 14 days of ensiling, regardless of LAB inoculation, as the yeast count increased (Li and Nishino, 2011b). There was no heating due to aerobic spoilage in the 56-day silage inoculated with *L. plantarum* and *L. buchneri*. Despite the improved preservation quality, Cai et al. (1999) discovered that the *L. plantarum* inoculation was ineffective at preventing yeast growth, resulting in aerobically deteriorated alfalfa, Italian ryegrass, and sorghum silages. However, Li et al. (2020) discovered that *L. plantarum*-producing class IIa bacteriocins improved alfalfa silage fermenting quality, reduced mold and yeast levels, and improved aerobic stability. Acetic acid promotes aerobic stability by inhibiting the growth of unfavorable bacteria such as molds and yeasts (Wilkinson and Davies, 2013). Therefore, the aerobic stability of the *L. buchneri*-treated silage of *L. chinensis* is likely.

**TABLE 3:** Fermentation products and microbial counts of wilted *Leymus chinesis* silage without and with *Lactiplantibacillus plantarum* (LP) or *Lentilactobacillus buchneri* (LB) and subjected to 14 and 56 days of preservation followed by 7-day AS (aerobic stability) test.

	14 days + AS				56 days + AS				2-way ANOVA		
	C	LP	LB	SE	C	LP	LB	SE	I	S	I × S
Dry matter (g/kg)	421b	439a	419b	4.47	436	431	441	6.27	NS	NS	*
pH	6.48b	6.88a	4.83c	0.09	6.85x	4.63y	4.54y	0.04	**	**	**
Lactic acid (g/kg DM)	2.48c	3.66b	12.8a	0.29	2.40z	16.4x	13.1y	0.24	**	**	**
Acetic acid (g/kg DM)	1.75b	2.19b	5.69a	0.13	1.63z	3.43y	4.86x	0.08	**	NS	**
Butyric acid (g/kg DM)	1.51a	0.21c	0.73b	0.06	1.61x	0.50z	0.84y	0.07	**	*	NS
Ethanol (g/kg DM)	0.26c	0.81b	1.55a	0.07	0.58xy	0.25y	0.77x	0.10	**	**	**
2,3-Butanediod (g/kg DM)	0.24c	0.64b	1.48a	0.06	0.68y	0.83y	3.43x	0.09	**	**	**
1,2-Propanediol (g/kg DM)	0.31b	0.43b	1.24a	0.10	0.46z	0.86y	1.49x	0.07	**	**	NS
Lactic acid bacteria (log cfu/g)	8.41b	9.39a	9.54a	0.09	8.42z	9.18y	9.80x	0.08	**	NS	NS
Yeasts (log cfu/g)	8.48	8.18	<2.00	–	8.46	<2.00	<2.00	–	–	–	–
Enterobacteria (log cfu/g)	7.27	<2.00	<2.00	–	7.39	<2.00	<2.00	–	–	–	–

Means from triplicate silages; and Values for identical preservation periods with varying following letters (a-c, x-z) differ significantly (P < 0.05). I and S represent the effects of inoculations and preservation periods, and I × S refers to the interaction of the two. **P < 0.01; *P < 0.05; and NS indicates P ≥ 0.05.

### Role of *Lactiplantibacillus plantarum* and *Lentilactobacillus buchneri* in microbial community diversities in ensiling and after aerobic exposure

Next-generation sequencing can provide detailed information on the microflora composition of whole crop corn, alfalfa, and sugarcane top silages (Guo et al., 2018; Hu et al., 2018; Wang et al., 2020b). However, there is currently no research on how homofermenter *L. plantarum* or heterofermenter *L. buchneri* affects dynamic changes in microbial communities in *L. chinensis* silage and after aerobic exposure. In Table 4, all samples had coverage values around 0.999, indicating that the majority of bacterial and fungal microflora were collected. For all samples, the 16S rRNA gene and the ITS gene were sequenced using high-throughput amplicon sequencing, yielding a total of 3,157,850 quality sequence reads. These readings were clustered into 8,935 OTUs with a dissimilarity of 3%. At 14 days, OTUs and Chao1 levels were higher after *L. buchneri* and *L. plantarum* inoculation than in the non-treated control, implying an increase in bacterial abundance with LAB inoculation compared to untreated silage. At 56 days, there was a reduction in OTUs and Chao1 compared to untreated silage after *L. buchneri* and *L. plantarum* inoculation. Guo et al. (2018) found that at 60 days, the levels of OTUs and Chao1 were higher in the LAB-inoculated alfalfa silage than in the non-inoculated samples, regardless of the fermentation method. On the 14th day, the Shannon index for bacterial diversity in *L. buchneri*-treated silage was the lowest, most likely due to microbial growth inhibition and subsequent bacterial diversity decline caused by lower pH in *L. chinensis* silage. The Chao, ACE, and number of OUTs decreased in spoilage silage after a 7-day aerobic deterioration test, indicating that aerobic microorganisms were not resistant to acids multiplied throughout the aerobic degradation test (Wilkinson and Davies, 2013). In addition, a denaturing gradient gel electrophoresis study revealed that when dominant bacteria become common in Italian ryegrass and alfalfa silages, the microbial community becomes less diverse (Li and Nishino, 2011a; Wu and Nishino, 2016).

**TABLE 4:** The bacterial and fungal alpha diversity of wilted *Leymus chinesis* silage during ensiling and aerobic stability (AS) test.

	Days	Sample ID	Read	OTU	Shannon	Chao1	ACE	Coverage
Bacterial alpha diversity	0 days	FM	193,130	621	3.923	691.187	693.744	0.999
	14 days	C	112,646	430	2.482	493.891	492.299	0.999
		LP	135,886	537	2.970	646.069	675.193	0.999
		LB	270,119	511	2.040	571.059	585.817	0.999
	14 days+AS	C	158,829	278	3.376	325.467	328.046	0.999
		LP	107,445	432	2.917	478.928	494.761	0.999
		LB	265,331	535	2.692	611.073	631.622	0.999
	56 days	C	235,586	729	3.297	791.880	808.282	0.999
		LP	207,850	322	1.151	404.234	412.852	0.999
		LB	184,490	327	1.435	396.102	401.845	0.999
	56 days+AS	C	116,550	379	3.070	430.262	440.859	0.999
		LP	160,147	399	0.954	458.450	474.308	0.999
		LB	216,995	331	1.948	379.212	383.662	0.999
Fungal alpha diversity	0 days	FM	61,556	68	0.501	75.167	75.726	0.999
	14 days	C	61,144	347	3.341	391.633	387.251	0.999
		LP	60,100	385	3.687	445.997	442.550	0.999
		LB	54,670	376	3.756	420.886	415.874	0.999
	14 days+AS	C	65,931	28	0.780	37.900	56.329	0.999
		LP	64,606	286	2.610	360.097	357.693	0.999
		LB	61,692	365	3.507	452.738	442.518	0.999
	56 days	C	51,698	191	3.293	220.989	205.738	0.999
		LP	65,969	318	3.313	355.564	344.533	0.999
		LB	55,251	144	3.645	157.444	158.167	0.999
	56 days+AS	C	67,851	24	0.668	35.000	43.245	0.999
		LP	62,482	245	2.314	280.143	300.006	0.999
		LB	59,851	327	3.186	368.193	352.611	0.999

During the aerobic deterioration test in *L. chinensis* silage, the richness and diversity of the fungi differed from that of the bacteria. The number of OTUs, Chao, and ACE indexes of fungi in untreated silage decreased after aerobic deterioration test than silo opening regardless of ensiling time, which was in accordance with Wang et al. (2020b). However, fewer changes in the fungi alpha diversity were observed in silage-inoculated *L. buchneri* on the 7-day aerobic test compared with silo opening at 14 days. One possible reason is that the high levels of acetic acid and 1,2-propanediol in silage-inoculated *L. buchneri* prevented certain fungi from dominating.

**FIGURE 1:**
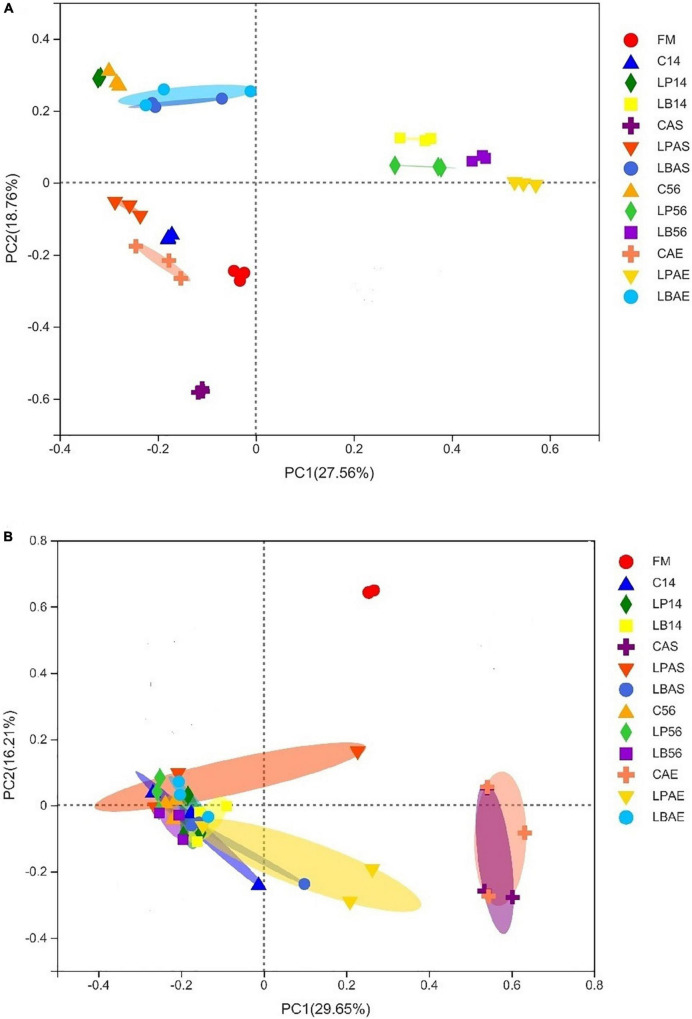
Principal coordinate analysis (PCoA) findings for the bacterial **(A)** and fungal **(B)** community in wilted *Leymus chinesis* silage without and with *L. plantarum* or *L. buchneri*. FM stands for fresh material; C stands for control; LP refers to the *L. plantarum* inoculation, and LB refers to the *L. buchneri* inoculation. The numbers following C, LP, and LB stand for the ensiling days. AS and AE of the following C, LP, and LB represent the aerobic stability test.

The principal coordinates the fungal and bacterial microflora analysis in wilted silages of *L. chinensis* inoculated with and without *L. plantarum* or *L. buchneri* were shown in Figure 1. As shown in Figure 1A, the inoculation of *L. plantarum* and *L. buchneri* affected the bacterial microflora of silage during ensiling. Similar results were observed in *Broussonetia papyrifera* leaves silage inoculated with *L. plantarum* (He et al., 2021). As the CAS, CAE, and LPAS samples were detected heating after aerobic exposure, they were separated from the other samples. There was a significant separation of fungal populations between fresh material and silages (Figure 1B). As fungal community changes in the CAS and CAE samples induced aerobic degradations, they were separated from the other samples.

### Roles of *Lactiplantibacillus plantarum* and *Lentilactobacillus buchneri* in microbial community dynamics in ensiling as well as after aerobic exposure

**FIGURE 2:**
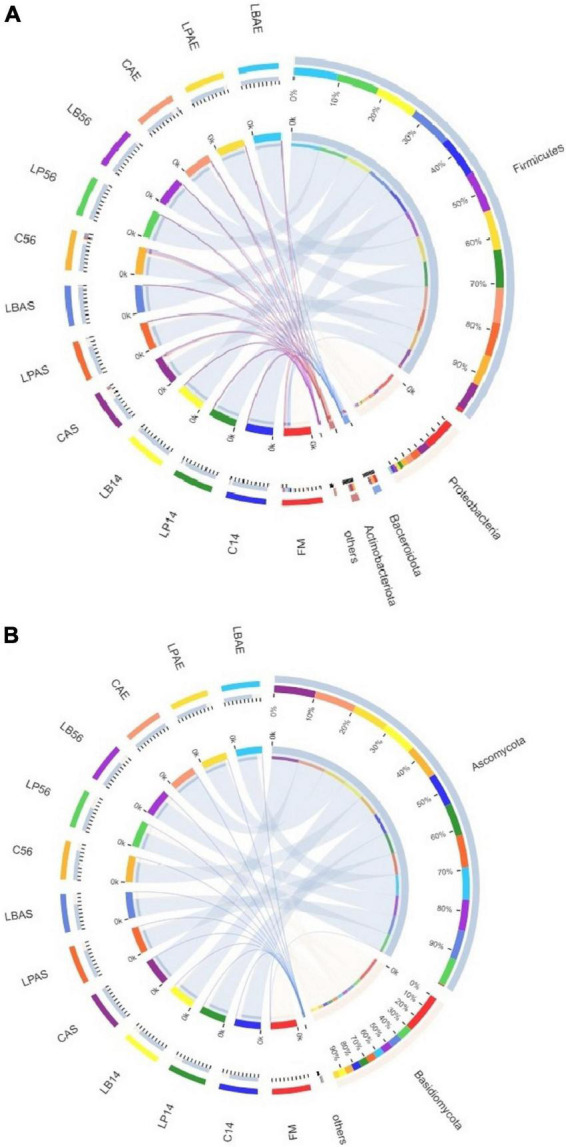
The phylum-level bacterial **(A)** and fungal **(B)** community in wilted *Leymus chinensis* silage inoculated with and without *L. plantarum* or *L. buchneri*. FM stands for fresh material; C stands for control; LP refers to the *L. plantarum* inoculation, and LB refers to the *L. buchneri* inoculation. The numbers following C, LP, and LB are the ensiling days. AS and AE of C, LP, and LB represent the aerobic stability test.

**FIGURE 3:**
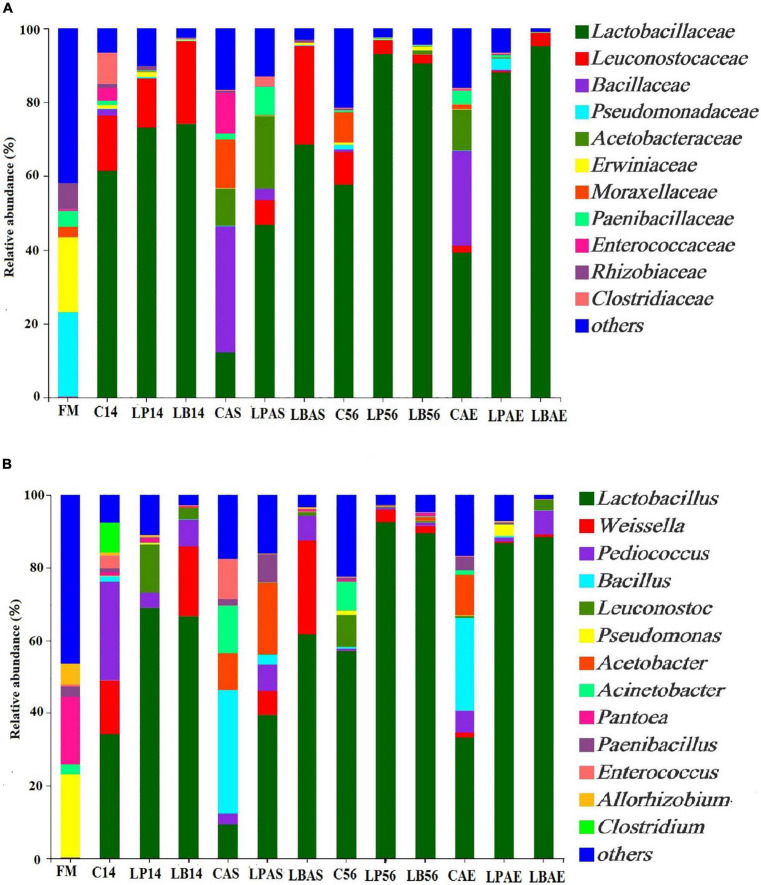
**(A)** Family- and **(B)** genus-level bacterial community in wilted *Leymus chinensis* silage inoculated with and without *L. plantarum* or *L. buchneri*. FM stands for fresh material; C stands for control; LP refers to the *L. plantarum* inoculation, and LB refers to the *L. buchneri* inoculation. The numbers following C, LP, and LB indicate the ensiling days. AS and AE following C, LP, and LB represent the aerobic stability test.

Figures 2A, 3A show the bacterial communities of pre-ensiled crop, silages, and after aerobic exposure at the phylum and family levels. *Proteobacteria*, *Firmicutes*, *Bacteroidota*, and *Actinobacteriota* were the most common phyla in pre-ensiled crops (Figure 2A). However, *Proteobacteria* and *Firmicutes* were the most common phyla in all samples after ensiling and aerobic exposure, accounting for more than 84.65% of all detected sequences, according to Figure 3A. The dominant families in pre-ensiled crops were *Pseudomonadaceae* and *Erwiniaceae*, which were found in fresh materials such as alfalfa, corn, and Italian ryegrass (Ni et al., 2017; Wang et al., 2020a). Despite the fact that their sections died during the ensiling process, *Lactobacillacae* and *Leuconostocaceae* emerged as the most dominant families after 14 and 56 days of ensiling, with or without LAB inoculants, respectively. Similarly, Ni et al. (2017) discovered a 70% overall *Lactobacillacae* and *Leuconostocaceae* ratio in Italian ryegrass silage after 60 days of ensiling. *Lactobacillacae* levels were higher in silage inoculated with *L. plantarum* and *L. buchneri* than in untreated silage at opening day-14 and -56 silos. Similar results were obtained in whole plant hybrid Pennisetum silage that was ensiled with *L. plantarum* (Shah et al., 2020). However, after aerobic exposure, the bacterial microflora composition of LAB-inoculated silages differed significantly from that of non-inoculated silages. The non-inoculated and *L. plantarum*-treated silages had higher *Acetobacteraceae* and *Bacillaceae* richness but lower *Lactobacillaceae* richness than the *L. buchneri*-treated silage in the 7-day spoilage test after opening day-14 silos. In the 7-day spoilage test after opening day-56 silos, untreated silage had more *Acetobacteraceae* and *Bacillaceae* and less *Lactobacillaceae* than LAB-inoculated silage. *Acetobacteraceae*, an organic acid-consuming bacterial species that occasionally causes aerobic deterioration, was discovered in silage by da Silva et al. (2020). Furthermore, after the start of spoiling, the *Bacillaceae*, which were generally present in the air-exposed silage, increased (Okatsu et al., 2019). After aerobic exposure, the *L. buchneri*-treated silage had lower *Acetobacteraceae* and *Bacillaceae* richness, as well as higher *Lactobacillaceae* richness, than the non-inoculated silage, regardless of ensiling process, implying that *L. buchneri* inoculation after aerobic exposure improves silage aerobic stability.

Figure 3B depicts the genus-level bacterial community of pre-ensiled crops, silages, and after aerobic exposure. Those predominant genera of pre-ensiled crops included *Pseudomonas*, *Pantoea*, *Acinetobacter*, *Paenibacillus*, and *Pedobacter*. Shah et al. (2020) reported similar results that *Pseudomonas* and *Pantoea* were dominated in fresh material of whole-plant hybrid Pennisetum. *Lactobacillus* was the most prevalent genus in every sample taken between the 14th and 56th days of ensiling, which is consistent with previous findings (Liu et al., 2019; Wang et al., 2020b). However, after aerobic exposure, the non-inoculated silage had higher *Acetobacteraceae* and *Bacillaceae* richness and lower *Lactobacillaceae* richness than the *L. buchneri*-treated silage. In the 7-day spoilage test after opening day-14 silos, the *Acetobacter* proportion increased from 0.32 to 19.66%, while the *Lactobacillus* proportion decreased from 68.99 to 39.41% in *L. plantarum*-inoculated silage. Therefore, these findings indicated that aerobic exposure decreased the *Lactobacillus* proportion in silage and that *L. plantarum* inoculation did not maintain relative *Lactobacillus* richness after silos were opened at 14 days. Following a 7-day aerobic exposure, ensiling with *L. buchneri* helped maintain high relative *Lactobacillus* richness (66.71% at 14 days, 89.52% at 56 days), implying that *L. buchneri* plays a *Lactobacillus*-enriching role in the silage. This was similar to what was discovered in alfalfa silage (Guo et al., 2018). In this study, once aerobic deterioration occurred, the amount of *Acetobacter* and *Bacillus* increased in untreated silage. *Acetobacter*, a non-fermenting aerobic bacterium, can be detected using denaturing gradient gel electrophoresis in whole crop corn silage (Li and Nishino, 2011b). The *Acinetobacter* bacteria were visible in all non-inoculated silage after 7 days of aerobic exposure, as well as in *L. plantarum*-treated silage after 7 days of aerobic exposure in 14 days of ensilage, though they did not grow in *L. buchneri*-inoculated silage after 7 days of aerobic exposure. Therefore, after a 7-day aerobic exposure, a high abundance of *Acinetobacter* promoted spoiling microbial development, reducing the aerobic stability of *L. chinensis* silage. Similar to *Acinetobacter*, *Bacillus*, and *Lysinibacillus*, increased aerobic species richness was observed in non-inoculated silage following aerobic spoilage, but not in *L. plantarum*-and *L. buchneri*-treated silages. S*erratia nematodiphila*, *Myroides odoratimimus*, *Lysinibacillus fusiformis*, *Stenotrophomonas maltophilia*, *Bacillus pumilus*, and *Acinetobacter soli* were discovered in the silage of corn stalk at the silo opening, and the aerobic bacteria were attributed as the likely cause of the aerobic spoilage (Liu et al., 2013).

**FIGURE 4:**
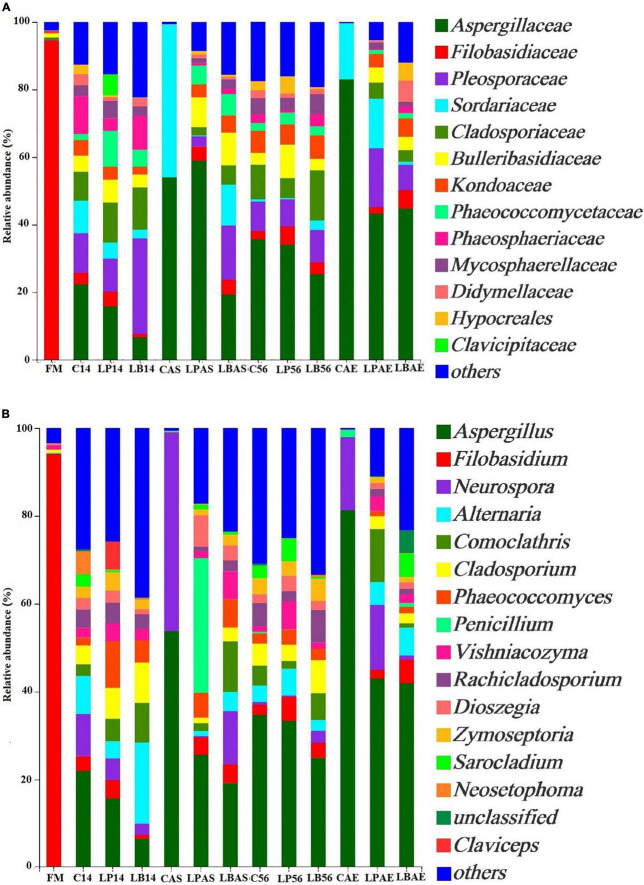
Fungal community at the family **(A)** and genus **(B)** levels in wilted *Leymus chinensis* silage without and with *L. plantarum* or *L. buchneri*. FM stands for fresh material; C stands for control; LP refers to the *L. plantarum* inoculation, and LB refers to the *L. buchneri* inoculation. The numbers following C, LP, and LB indicate the ensiling days. AS and AE following C, LP, and LB represent the aerobic stability test.

Figures 2B, 4A show the fungal population of the pre-ensiled crop, silages, and after aerobic exposure at the phylum and family levels. *Ascomycota* and *Basidiomycota* were the most common phyla of pre-ensiled crops and silages, respectively (Figure 2B). Following aerobic exposure, the dominant phylum in CAS and CAE samples was *Ascomycota* alone, accounting for 99.89% of all sequences found. The *Filobasidiaceae* family was the most abundant in the pre-ensiled crop, accounting for more than 94.45% of total abundance (Figure 4A). However, the *Aspergillaceae*, *Pleosporaceae*, *Cadosporiacaeae*, and *Bulleribasidiaceae* were the dominant families in every sample after ensilage, accounting for more than half of all detected sequences. After aerobic exposure, changes in the fungal microflora were observed in the non-inoculated silage, with *Aspergillaceae* and *Sordariaceae* emerging as the dominant families, accounting for more than 97% of the total abundance. Fungi were also found in the corn silage, but there was no evidence of *Sordariaceae* involvement in the aerobic deterioration (Sandona et al., 2019). Mycotoxins, according to Gallo et al. (2021), are a group of secondary metabolites produced by *Aspergillaceae* during aerobic exposure to corn silage. Therefore, secondary fermentation was most likely triggered in the non-inoculated silage. When compared to 7 days after aerobic exposure, the changes in fungal microflora in *L. buchneri*-treated silage were insignificant at silo opening. The total amount of yeast after aerobic exposure of *L. buchneri*-inoculated silage was less than 10^2^ CFU/g, which could explain why the silage did not heat after 7 days of aerobic exposure. Furthermore, Wang et al. (2020b) discovered that the total amount of yeasts in *Lactobacillus hilgardii*-inoculated silage was lower than in untreated silage, which could explain why the pH and temperature of the silage treated with *L. hilgardii* did not reach their maximums after aerobic exposure. Figure 4B depicts the fungal population of pre-ensiled crop, silage, and after aerobic exposure at the genus level. *Filobasidium* was the most common genus in the fungal microflora of the pre-ensiled crop, appearing in nearly 94% of the sequences. The findings of Zhang et al. (2021) were also consistent, with *Filobasidium* being found to be the richest fungus in the fresh wheat sample. Despite the fact that *Aspergillus*, *Filobasidium*, and *Penicillium* were the most common fungi discovered in this study, *Aspergillus* was the most common fungus at the genus level after 56 days of silage preservation. After aerobic exposure, the proportion of *Aspergillus* was higher than at silo opening in untreated silage. However, after aerobic exposure, the proportion of *Aspergillus* in the *L. buchneri*-treated silage was lower than in the non-inoculated silage because LAB (heterofermentative) inoculation into the silage of *L. chinensis* likely inhibited the aerobic deterioration caused by fungi post-spoilage growth. This finding contradicts the findings of Wang et al. (2020b). Fungal spoilage of animal feed silage is common during feed-out. *Aspergillus fumigatus* and *Penicillium roqueforti* were the most common spoilage molds in sugar beet press pulp and maize silage (Nout et al., 1993). The proportion of *Penicillium* was higher after aerobic exposure than after 14 days of ensiling with *L. plantarum*-inoculated silage in this study. Therefore, this could explain why heating was detected on the third day after silo opening for *L. plantarum*-inoculated silage.

### Correlation analysis of the microbial community with fermentation products

**FIGURE 5:**
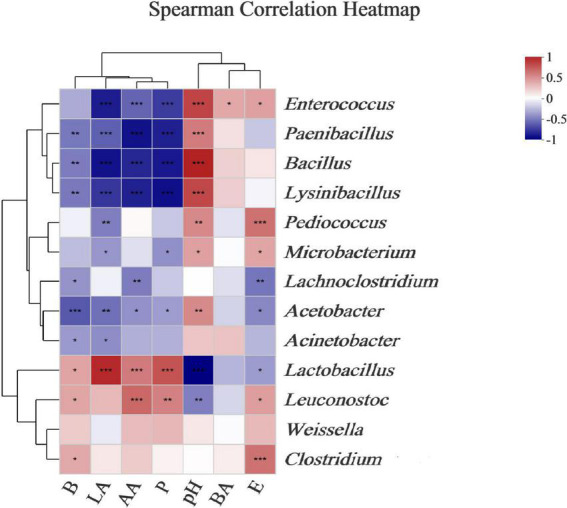
Correlations of bacterial richness with the fermented products. Heatmaps analyzing Spearman’s rho, as well as *p*-values for the pairwise comparisons of bacterial genera and fermented products. *, 0.01 < *P* < 0.05; **, 0.001 < *P* < 0.01; and ***, *P* < 0.001. LA, AA, and BA, respectively, stand for the lactic, acetic, and butyric acids; E, Ethanol; B, 2,3-Butanediod; P, 1,2-Propanediol.

**FIGURE 6:**
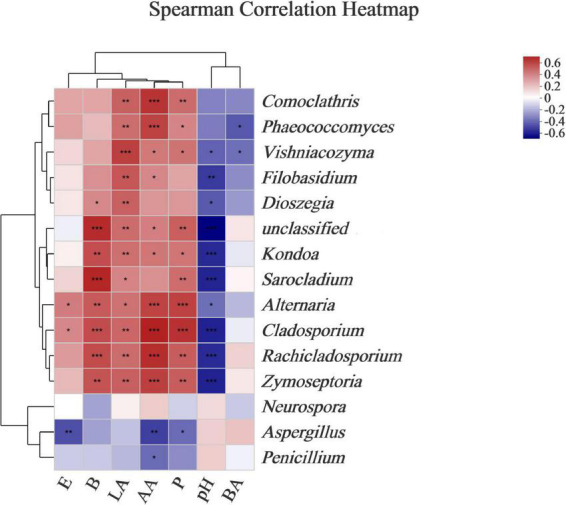
Correlations of fungal richness with the fermented products. Heatmaps analyzing Spearman’s rho, as well as *p*-values for the pairwise comparisons of fungal genera and fermented products. *, 0.01 < *P* < 0.05; **, 0.001 < *P* < 0.01; and ***, *P* < 0.001. LA, AA, and BA, respectively, stand for the lactic, acetic, and butyric acids; E, Ethanol; B, 2,3-Butanediod; P, 1,2-Propanediol.

Ensilage and aerobic exposure were both complex processes involving interactions between various fermentation products and microflora. Beneficial bacteria reduce aerobic deterioration by producing a variety of metabolites, in addition to improving fermentation quality. For example, LAB-producing class IIa bacteriocins could improve silage fermentation quality, lower mold and yeast counts, and improve aerobic stability (Li et al., 2020). Mold has been linked to mycotoxin production, whereas enterobacteria can ferment WSCs into products such as lactic and acetic acids (Mcdonald et al., 1991). To better understand the correlations between microbial dynamics and fermented products (e.g., acetic acid, lactic acid, butyric acid, ethanol, 2,3-butanediol, and 1,2-propanediol) during *L. chinensis* ensilage, and following aerobic exposure, a genus-level heatmap of the Spearman correlation was plotted for the major bacteria and fungi (Figures 5, 6). The *Lactobacillus* and *Leuconostoc* genera had positive relationships with acetate acid, 2,3-butanediol, and 1,2-propanediol concentrations, according to the Spearman correlation analysis (Figure 5). In contrast, the *Paenibacillus*, *Bacillus*, *Lysinibacillus*, and *Acetobacter* genera had no relationships with acetate acid, 2,3-butanediol, or 1,2-propanediol levels. Lactic acid content was found to be positively correlated with the *Lactobacillus* genus (*R* = 0.88), whereas *Enterococcus*, *Pediococcus*, and *Acetobacter* richness were found to be negatively correlated with lactic acid, with correlation values of –0.79, –0.44, and –0.50, respectively. The relationship between lactic acid and bacterial richness can be described in a variety of ways based on the above findings. Despite the fact that lactic acid-producing cocci (e.g., *Leuconostocs* and *Pediococcus*) began lactic fermentation early in the ensilage stage, these bacteria were unable to survive at low pH. The *Lactobacillus* bacterium produces lactic acid in silage and has a significant impact on pH reduction during the late ensilage stage (Cai et al., 1998). Wang et al. (2020b) discovered a positive relationship between silage lactic acid content and *Lactobacillus* richness. Silage is deemed undesirable when *Acetobacter* spp. is present due to its ability to cause aerobic deterioration as well as acetic and lactic acid breakdown, resulting in CO2 and water production (Li and Nishino, 2011c). The *Kondoa*, *Alternaria*, *Cladosporium*, *Rachicladosporium*, and *Zymoseptoria* genera were positively correlated with lactic acid, 2,3-butanediol, and 1,2-propanediol concentrations, according to the Spearman correlation analysis (Figure 6). The acetate acid and the richness of *Comoclathris*, *Phaeococcomyces*, *Alternaria*, *Cladosporium*, *Rachicladosporium*, and *Zymoseptoria* were found to have positive correlation factors of 0.60, 0.56, 0.55, 0.65, 0.61, and 0.56. In contrast, negative relationships of acetate acid contents with the *Aspergillus* (*R* = –0.48) and *Penicillium* (*R* = –0.37) genera were discovered. Although Aspergillus has been shown to inhibit acetic acid fermentation, no studies have demonstrated that it has a significant impact on acetic acid reduction. In this study, Aspergillus, a common mold that produces mycotoxin in corn silages (Gallo et al., 2021), was found to have high richness after aerobic exposure to untreated silage. In the early stages of silage production, *Alternaria* and *Cladosporium* were discovered (Liu et al., 2019). There is less information on the fungal microbiota during the ensilage process and after aerobic exposure. Therefore, the roles of these fungi during forage ensilage, followed by aerobic exposure, are unknown.

## Conclusion

During ensiling, silages inoculated with *L. plantarum* and *L. buchneri* had higher lactic acid and LAB counts and lower pH, butyric acid, ethanol, and 2,3-butanediol contents. Heating was observed on the third day after silo opening for *L. plantarum*-treated and non-inoculated silages, which were opened on the 14th day. However, no heating was observed after silo opening in *L. buchneri*-inoculated silages. In the 7-day spoilage test after opening 56-day silos, silages inoculated with *L. plantarum* and *L. buchneri* had higher *Lactobacillus* richness and lower hazardous microorganisms, namely, *Acetobacter*, *Bacillus*, *Aspergillus*, and *Penicillium*, which were linked to silage deterioration. The composition of fermentation products was related to the bacterial community, particularly *Lactobacillus*, *Enterococcus*, and *Acetobacter*.

## Data availability statement

The raw data supporting the conclusions of this article will be made available by the authors, without undue reservation.

## Author contributions

BW and HN designed the experiment. ZH, MW, and MY conducted the experiments. ZH, MW, MY, and HN analyzed the data. BW wrote the manuscript. All authors read and approved the manuscript.
